# The Long Term Impact of Micronutrient Supplementation during Infancy on Cognition and Executive Function Performance in Pre-School Children

**DOI:** 10.3390/nu7085302

**Published:** 2015-08-07

**Authors:** Marisol Warthon-Medina, Pamela Qualter, Nelly Zavaleta, Stephanie Dillon, Fabiola Lazarte, Nicola M. Lowe

**Affiliations:** 1 International Institute of Nutritional Sciences and Applied Food Safety Studies, University of Central Lancashire, Preston PR1 2HE, UK; E-Mails: MWarthon-Medina1@uclan.ac.uk (M.W.-M.); SDillon@uclan.ac.uk (S.D.); 2 School of Psychology, University of Central Lancashire, Preston PR1 2HE, UK; E-Mail: PQualter@uclan.ac.uk; 3 Instituto de Investigación Nutricional, Lima, Peru (IIN), Avenida La Universidad, La Molina 1885, Lima 12, Peru; E-Mails: nzavalet@iin.sld.pe (N.Z.); fabiolalazarte@hotmail.com (F.L.)

**Keywords:** zinc, iron, multiple micronutrient supplementation, cognitive function

## Abstract

Brain growth and development are critically dependent on several micronutrients. During early development cellular activity may be sensitive to micronutrient deficiencies, however the evidence from human studies is equivocal. The objective of this study was to examine the long-term cognitive and social-emotional effects of multiple micronutrient supplementation compared with iron supplementation alone, administered during infancy. This study was a follow-up to an initial randomized, double-blind controlled trial (RCT) in 2010 in which 902 infants, aged 6–17 months, from Lima, Peru, were given daily supplements of either iron (Fe) or multiple micronutrients (MMN) including zinc (451 in each group). The supplementation period for both groups was six months. In 2012, a subsample of 184 children from the original cohort (now aged 36–48 months) was randomly selected to participate in a follow-up trial and was assessed for intelligence, working memory, inhibition, and executive function. The tests showed no significant differences between the supplementation groups though there were some gender differences, with girls displaying higher scores than boys across both groups on the Wechsler Preschool and Primary Scale of Intelligence (WPPSI) Verbal IQ sentences subtest, the Day-Night cognitive test and on the Brief Infant-Toddler Social Emotional Assessment (BITSEA) social competency, and boys scoring higher than girls in problem behaviour. The results indicate that MMN supplementation had no long term additional effects on cognitive function compared with iron supplementation alone. The timing of supplement administration for maximum impact on a child’s cognitive development requires further investigation.

## 1. Introduction

Malnutrition is a worldwide problem that affects millions of unborn and young children mainly during the vulnerable stages of brain development [1]. There is also substantial evidence that severe malnutrition in early childhood can lead to poor cognitive function and delayed child development [2]. Intellectual function appears to be impaired by malnutrition during childhood [3], and often is associated with iodine, iron, zinc, folic acid, and vitamin B12 deficiencies [4]. A review by Olson *et.al*. [5] indicates that vitamin A maintains neuronal plasticity and cognitive function, and has an important role in learning and memory processes which are related to the function of the hippocampus [6]. Folate may also contribute to brain health due to its role as a cofactor in one-carbon metabolism [7]. Harrison *et al.* [8], indicates that vitamin C is essential for neuronal maturation and function and has a protective role of the brain against oxidative stress. All of these micronutrients are important for post-natal brain development and have a critical role in brain nutrition, maintaining its structure and functionality [9,10,11].

Furthermore, there is fundamental evidence in the literature that micronutrients are essential for cognitive development; the interventional study by Muthayya *et al*., showed that higher micronutrient treatment with iodine, iron, riboflavin, vitamin B6, vitamin B12, folate and vitamin A in Indian children aged 6–10 years old, improved short term memory [12]. In addition, the review by Eilander *et al*., indicated a possible association between micronutrients, a small increase in fluid intelligence (the ability to reason and think abstractly) and academic performance [13].

Iron and zinc deficiency remain a global health problem, particularly in children from developing countries where malnutrition is likely [14]. In addition, nutritional deficiencies often occur in the presence of economic disadvantage and poor physical resources [15].

Nutrients are very important during early life for neuronal cell growth, therefore, malnutrition impacts brain development [16] and, in turn, can also impact on emotions and behaviour. So, under-nutrition is associated with children’s poor cognitive, behavioural development, and intellectual impairment in later life, but polyunsaturated fatty acids (PUFA), particularly PUFA n-6, have also been shown to play an important role in the development of the theory of mind [17].

Pathways linking deficiency of zinc to poor cognitive function and delayed child development include the interference of the formation of neural pathways and cellular neurotransmission [18]. Children who are zinc deficient are also likely to be iron and B12 deficient because the main source for all of these micronutrients is animal protein [19], The preschool years (1 to 5 years of age) are a period of rapid and dramatic post-natal brain development, and the main period of cognitive development, with increases in working memory, attention and inhibitory control [20,21], therefore ensuring good micronutrient status during this period is essential.

Human studies have shown that the effect of malnutrition on psychological development is dependent on the degree of malnutrition that can be categorized as moderate or severe [22,23]. A review by Grantham-McGregor and Baker-Henningham [24], suggest that a well-balanced diet is necessary for optimal development in children and considerable evidence exists that the first two years of life are the most sensitive to under-nutrition. Another review by Morgane states that malnutrition exerts its effects during development, not only during the brain growth spurt period, but also during early organizational processes such as neurogenesis, cell migration, and differentiation [25]. The preschool years are also a time of transition from a direct maternal mediation/selection of diet-based nutrition to food selection that is based more on self-selection and self-gratification. However, the majority of published studies examining the role of nutrition in cognitive function are in infants or school-aged children, with very few studies investigating preschool children [21].

Evidence from observational studies suggests that micronutrients may play an important role in the cognitive development of children, but the results of intervention trials using single micronutrients are inconclusive. In addition, there is a paucity of data regarding the impact of multiple micronutrient supplementation on cognitive development.

Focusing on malnutrition, and suitability of population for the current study, Peru is a country considered at high risk of zinc deficiency [26] and of iron deficiency anaemia. The prevalence of anaemia in Peruvian children aged 6–59 months is on average 43%, while in women this accounts for 32.2% [27]. Furthermore, child chronic malnutrition remains one of the main public health problems in Peru with a prevalence of 19.5% in children under 5 years old [28]. Interventions with MMN in Peru have shown to be effective in reducing anaemia prevalence [29,30,31]. As a result Peru was chosen as the location for this study. Therefore, the aim of the current study was to elucidate the following research question: Does multiple micronutrient supplementation at 6 months of age have long term effects on children’s cognitive and social-emotional skills, compared with iron supplementation alone?

## 2. Methods

### 2.1. Study Design

In order to investigate the long-term effects of supplementation this paper presents a follow-up to an original study. The original study was conducted in 2010, funded by the United Nations Children’s Fund (UNICEF) and conducted by the Instituto de Investigación Nutricional (IIN), Lima, Perú. A population of 902 infants, aged between 6 and 17 months, were randomized to receive daily powdered supplements of (a). 12.5 mg iron (control) *vs.* (b) multiple micronutrients containing 12.5 mg iron, 10 mg zinc gluconate, 160 μg folic acid, 30 mg vitamin C, and 300 μg vitamin A. The supplements, provided in the form of sprinkles (single dose sachets, like small packets of sugar) [32], were easily sprinkled onto foods prepared in the household [33]. The supplementation in the initial study [34] was for a period of 6 months. The district of Villa El Salvador was selected for this study because a previous food consumption study [35,36,37] indicated that people in this district were at risk of anaemia and micronutrient deficiency due to a low consumption of zinc-rich foods.

The follow-up study presented here included the same population in Lima, Perú. The study was conducted in July–September 2012 (the winter season in Peru), when children were 36–48 months old. Assuming a power of 80%, and a confidence level of 95%, for the verbal and full scale IQ cognitive outcome, the number of participants required was 166 children in total; 10% was added for the loss of participants across the different sessions where cognitive tests were administered [38,39], giving 182 children in total. The Institutional Review Boards of the Instituto de Investigación Nutricional (IIN) and the University of Central Lancashire (UCLAN) approved the study protocol 335-2012/CEI-IIN on the 4th of July, 2012.

For this study, the hypothesis to be tested was multiple micronutrient supplementation during infancy has a greater impact on cognitive function at pre-school age than iron alone.

### 2.2. Participant Recruitment

**Figure 1:**
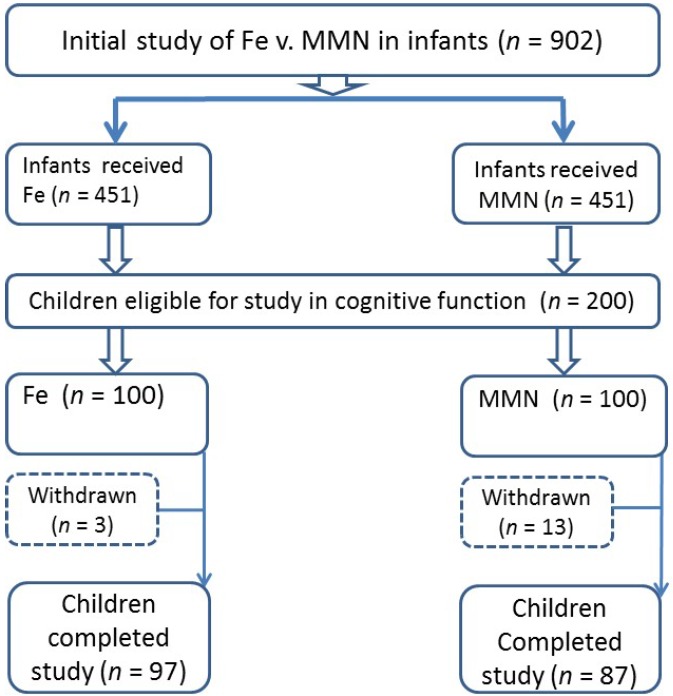
Study design.

200 children were randomly selected form the original study cohort. The families were visited by two health workers to invite them to participate in the pre-school study. The process was repeated (random selection and visit) until a total of 200 were recruited. Parental consent was obtained for 184 children to participate in the study. However, due to absences across the assessments, complete data were only available for 182 children for the majority of tests (see Figure 1). Mothers’ ages ranged from 18 to 48 years. (mean ± SD, 31 ± 6.46 year) at the time of the current study. At the time of cognitive and social-emotional test administration, the children’s ages ranged from 36 to 48 months (mean ± SD, 41.48 ± 3.19 months). The two groups were identified as “Iron (Fe)” (*n* = 97), and “Multi-Micronutrients (MMN)” (*n* = 87). Group allocation remained blinded to the research team throughout the data collection and analysis phases.

### 2.3. Study Methodology

Evaluation of cognitive functioning involved the assessment of intelligence, working memory, inhibition, and executive function (attention). To obtain a measure of each child’s overall cognitive development the Wechsler Preschool and Primary Scale of Intelligence (WPPSI) tests [40] were administered. WPPSI had previously been used in Peruvian children to measure the child’s Intelligence Quotient (IQ) and had been validated [38,41,42]. Additionally, a Spanish version of the test was available at IIN, so it was selected for use.

The Day-Night Stroop test [43] was used to evaluate cognitive flexibility and processing speed, measuring inhibitory control, namely the ability to suppress a dominant automatic response to a stimulus [44]. This ability to inhibit a dominant response improves with age [45]. Inhibition also involves interference control, directed forgetting, emotional control, and motor control [46]. Inhibition plays an important role in determining how mental processes are involved and work together in executing a task [47]. The Day-Night Stroop test has previously been used in other studies for children aged 3.5 to 7 years [43,48]. This test has also been used in developing countries, but not widely [49]; we, therefore, ensured appropriate use of the current sample by way of a pilot study by IIN.

To measure working memory, the nine boxes test was used [48]. This test exercised the working memory and attention of the child and activated part of the memory system involving use of short-term memory, in the prefrontal cortex [50]. Short-term memory is defined as a type of memory used to retain information for a short time that includes the working memory component, which is a type of mental workspace or sketchpad in the mind that is used to manipulate information in consciousness [51]. Working memory is a more complex construct than short-term memory [52] and its measurement is important as it plays a causal role in children’s developing skills and knowledge, mainly in the area of literacy [53].

To measure state of reasoning, the Theory of Mind (false belief task) [54] was used. The theory of mind is the ability to attribute mental states to oneself and others [55]. The complexity of the theory of mind requires that children must be able to understand the relation between an object and a person’s percept which is conditional on a third variable, and the processing of three interactive variables is often difficult for young children [56]. Thus, conflicting ideas need to be understood concurrently [57]. The test involves the unexpected transfer of an object, so that the protagonist then has a false belief about the location of that object.

To measure the child’s social-emotional development the Brief Infant-Toddler Social Emotional Assessment (BITSEA) [58] standardized parent questionnaire was used. Briggs-Gowan, *et al.* [59] suggests that the BITSEA test may be a valuable tool to aid screening, identification, and assessment of early social-emotional/behavioural problems. This tool has been shown to be a valid screening method for identifying social emotional and behavioural problems and/or delays or deficits in social-emotional competence in children aged from 12–35 months [60]. In our study, some children were over 36 months old and consequently, a new cut-off point for children over 35 months of age was determined. Standardized values were created from mean scores of problem total from the BITSEA questionnaire. The cut-off points were set at 1 standard deviation above the mean. Data were transformed and recoded with variable labelled as 1 for children with BITSEA problem, and 2 for children without BITSEA problem. The cut off point for problem was set at 1 standard deviation (1), above the mean and the cut off point for good level of competence was set at 1 standard deviation (−1) below the mean. Moreover, to ensure appropriate use, the BITSEA test was trialed in a pilot study (*n* = 13) at the Villa El Salvador Clinic in June–July 2012 as a forerunner to use in this study.

### 2.4. Test Procedure for Executive Function Cognitive Outcome

#### 2.4.1. Day-Night Stroop Test

For this test children needed to hold two rules in mind: they were instructed to say “day” when shown a black card with a picture of a white moon and stars, and to say “night” when shown a white card with a picture of a yellow sun. Pictures were on cards that were 20 cm long by 14 cm wide as used in previous studies (Gerstadt, Hong and Diamond [43],Carlson [61]). In the practice session, children practiced with four pictures (two pictures of the sun and two pictures of the moon). In the actual test session, children saw eight pictures of the sun and eight pictures of the moon, in a pre-determined order. Children’s responses on each trial of the Day-Night Task were scored as correct or incorrect, and provided total scores that could range from 0 to 16. In addition, we measured the longest run of consecutive correct answers.

#### 2.4.2. Nine Boxes

This was a memory task that involved the use of multiple boxes 6 cm by 6 cm with distinctive figures drawn on the lids, so that the children could differentiate one box from the other, and with a toy animal inside each. Boxes were positioned 6 cm apart approximately. The aim of the task was to open all boxes without repeating a choice, the boxes were scrambled or shuffled after each selection behind a screen. Hence, the child had to select a box that had not been opened before and take the toy animal.

The administration of this test started with three training boxes so that the children could get familiar with the test. Then, the test was started with nine boxes following the above procedure. The exercise was repeated and the child had a maximum of 20 chances to achieve the collection of nine animals from the nine boxes. A score of 1 was given if the child opened a box and took the animal from the box (full box); if the child opened a box that had no animal in it (empty box), the score given was zero. If five consecutive errors were made the test was discontinued. The child’s overall score was determined as the number of correct answers divided by the total number of attempts. In addition, we measured the longest run of consecutive correct answers.

#### 2.4.3. Theory of Mind

Materials used were two dolls (Maria and Rosa), two cupboards (one red and one blue), a chocolate, and a playground, based on the test as described in [54]. This false-belief task involves the unexpected transfer of an object, so that the protagonist then has a false belief about the location of that object. In this standard false belief story, children saw a character (Maria) place a chocolate in one of the cupboards (green) and then leave to play in the park. Another character (Rosita) then moved the chocolate to another cupboard (blue). When the original character returned (Maria) the children answered the experimental questions: (1) ‘‘Now when Maria comes back into the kitchen, where will she look for her chocolate?” (2) “Why does Maria look in that particular place for her chocolate?” The second question is asked to obtain an explanation to the answer to the first question and to corroborate whether the child understood that somebody can have a different opinion than him or her. To generate this conflicting response the child must use theory of mind knowledge to reason that Maria believes that the chocolate is in the green cupboard because she did not see it moved by Rosita to the blue cupboard. Language ability and verbal memory are important to predict false belief understanding and are underlying of mind development [62,63]. Between the ages of 3 to 5 years old, important changes occur in the development of the theory of mind, children at 3 year-old appear to have developed an understanding of their own false belief before they have developed a better understanding of other’s false belief, whereas children at 4.6 year-old seem to have a better understanding of false belief [64]. In the current study, children’s response was measured through the creation of an aggregate score for theory of mind, which was created by summing the score of questions 1 and 2, giving a range of scores from 0 to 2.

#### 2.4.4. BITSEA

This questionnaire contains 42 questions about the child’s behaviour, scored as 0, 1 or 2 (never occurs, sometimes occurs, always occurs respectively) and yields two scores for competence and problems. Mothers completed the questionnaire. In the BITSEA, there are six behavioural dimensions, as follows: (1) externalizing problems (difficulties with activity/impulsivity, aggression/defiance and peer aggression), (2) internalizing problems (fearfulness, worry, nervousness, distress upon separation, anxiety and social withdrawal), (3) dysregulation problems (negative emotionality, sleep, eating and sensory sensitivities), (4) competency (follow rules, attention skills, mastery motivation, imitation/play behaviour, pro-social interaction with peers and emerging empathy), (5) autism spectrum disorder items (repetitive behaviours, put things in a special order over and over, social competence and attention), and (6) “red flag” items (behaviour that may indicate clinically significant problem such as runs away in public places, hurts self on purpose or gags or chokes on food) [58]. Two forms of reliability, test-retest and inter-rater reliability are reported for the BITSEA form. The BITSEA form demonstrated strong one-year stability for the Problem total score (*r* = 65) and the Competence total score (*r* = 0.53), then both the BITSEA Problem total score and the BITSEA Competence total score have shown to have good retest reliability and good inter-rater reliability, therefore BITSEA is a reliable measure of children social-emotional problems and competencies [58]. Each subscale included three to six domains, for instance for externalizing (activity/impulsivity, aggression/defiance, peer aggression), for internalizing (depression/withdrawal, general anxiety, separation distress, inhibition to novelty), for dysregulation (negative emotionality, sleep, eating, sensory sensitivity), for competence (compliance, attention, mastery motivation, imitation/play, empathy and prosocial peer relations) [58].

### 2.5. Test Procedure for Intelligence Testing

#### WPPSI

The executive and verbal scales of the Wechsler Primary and Preschool Scales of Intelligence (WPPSI) were used to measure IQ. There were four subtests in the executive or performance scale (object assembly, block design, picture completion, animal pegs) and five subtests in the verbal scale (vocabulary, arithmetic, similarities, comprehension, and sentences). Wechsler Preschool and Primary Scale of Intelligence-Revised (WPPSI-R) subtests were chosen to measure variables of interest such as memory and attention. Selected subtests mentioned above were administered for a prorated overall IQ score.

### 2.6. Statistical Analysis Plan

Data analyses were completed using SPSS version 21.0 (IBM software products, Somers, NY, USA). Descriptive statistics were used to describe cognitive measurements of intelligence, memory and behaviour, with a number of statistical tests used to examine differences between the two groups of children who had been given supplementation. For all tests used, results with a value of *p* < 0.01 (two-tailed) were considered statistically significant, in line with Bonferroni’s correction [65].

For the WPPSI test of intelligence, a series of 2 (supplementation group) × 2 (gender) ANOVAs were used. They looked at verbal and executive subtests, and the overall intelligent quotient (IQ) scale. For the Day/Night, nine boxes, and theory of mind tests, 2 (supplementation group) × 2 (gender) ANOVAs were conducted to compare differences between the iron and the MMN groups on inhibition, working memory, and understanding of mental states respectively. For the BITSEA test, a series of 2 (supplementation group) × 2 (gender) ANOVAs were conducted to compare differences between the iron and the MMN groups on problem and competence reports from mothers. To examine whether the number and percentage of children in each group who presented problem behaviour and good levels of competence was different a χ² test (chi-square test), was used. For this, a total of four chi-squares (χ²) were conducted to determine the number of boys and girls who presented/did not present problem behaviour and the number of boys and girls that presented good/poor levels of competence. To examine parent’s response about their concern regarding language development and behaviour of their children, a series of 2 (supplementation group) × 2 (gender) ANOVAs were conducted on both language concern and behaviour concern to compare differences between the iron and the MMN groups.

## 3. Results

The characteristics of the study population are shown in Table 1 by group and gender, showing no significant differences between the two supplementation groups, or gender in terms of height or weight.

### 3.1. Cognitive Tests

#### 3.1.1. Day-Night Stroop Test

For the Day-Night Stroop 16 trials test inhibitory task, the 2 × 2 ANOVA did not reveal a main effect of groups (*F(1, 95)* = 0.006, *p* = 0.94). There was a significant gender main effect, (*F(1, 94)* = 6.749, *p* = 0.01), with girls having higher scores than boys in both groups for the longest run of consecutive correct answers for those who completed the 16 trial task of the Day-Night Stroop test (see Table 2). ANOVA revealed no significant group × gender interaction between on the Day-Night Test (*F(1, 94)* = 0.087, *p* = 0.77).

**Table 1:** Characteristics of the study population.

Variable	Fe (*n* = 97)			MMN (*n* = 87)			*p* value
	$\bar{\text{x}}$ ± SD	Min.	Max.	$\bar{\text{x}}$ ± SD	Min.	Max.	
**Age (months)**							
**All children**	**41.55 ± 3.16**	**36.07**	**47.87**	**41.40 ± 3.23**	**36.03**	**47.93**	**0.22**
Boys	41.65 ± 3.13	36.07	47.87	40.99 ± 3.04	36.03	47.38	
Girls	41.42 ± 3.23	36.95	47.15	41.92 ± 3.42	36.36	47.93	
**Weight (kg)**							
**All children**	**15.49 ± 2.14**	**11.5**	**22.5**	**15.41 ± 2.01**	**12**	**21.6**	**0.60**
Boys	15.59 ± 2.02	12.4	21.7	15.71 ± 1.98	12	21.6	
Girls	15.37 ± 2.29	11.5	22.5	15.02 ±2.02	12.4	19.7	
**Height (cm)**							
**All children**	**97.43 ± 3.64**	**90.8**	**107.5**	**97.16 ± 3.94**	**89**	**105.2**	**0.66**
Boys	97.9 ± 3.45	91.3	105.2	97.49 ± 3.83	91	105.2	
Girls	96.85 ± 3.81	90.8	107.5	96.74 ± 4.09	89	104.2	

**Table 2:** Inhibition (Day-Night Stroop test), Working memory (9 boxes) and reasoning (Theory of Mind, false belief task) outcomes by group, and gender.

	Fe	MMN	*p* value	Eta squared η^2^	Cohen’s *d*	Effect size *r*
	$\bar{\text{x}}$ ± SD	$\bar{\text{x}}$ ± SD				
**Longest correct run for Day-Night test ^†^**						
**All children**	**7.75 ± 4.79**	**7.98 ± 4.96**	**0.73**	**0.0001**	**−0.02**	**−0.009**
Boys	6.76 ± 4.35 *	6.81 ± 4.39 *	0.01 *	1.04		
Girls	9 ± 5.12 *	9.63 ± 5.37 *				
**Longest correct run for nine boxes test**						
**All children**	**4.46 ± 2.05**	**4.77 ± 2.20**	**0.26**	**0.007**	**−0.15**	**−0.07**
Boys	4.64 ± 2.11	4.57 ± 2.38	0.92	0.00006		
Girls	4.25 ± 1.89	5.03 ± 1.95				
**Aggregate score for the Theory of Mind (false belief task)**						
**All children**	**0.20 ± 0.49**	**0.25 ± 0.55**	**0.38**	**0.004**	**−0.12**	**−0.06**
Boys	0.21 ± 0.49	0.18 ± 0.49	0.39	0.004		
Girls	0.18 ± 0.50	0.34 ± 0.63				

**^†^** Longest correct run for Day-Night test for those who completed the 16 trial Day-Night test Fe (*n =* 52), MMN (*n =* 46); *F* (*1, 94*) = 0.125, *p* = 0.73; * significant differences by gender, *F* (*1, 94*) = 6.749, *p* = 0.01. Percentage of correctness (correct/attempts) for Day-Night test for those who completed the 16 trial Day-Night test Fe *n =* 53 (76.42%), MMN *n =* 46 (76.77%), *F* (*1, 95*) = 0.006, *p* = 0.94. Longest correct run for nine boxes test Fe (*n =* 97), MMN (*n =* 87); no effect by group *F* (1, 180) = 1.272, *p* = 0.26, non-significant effect by gender *F(1, 180*) = 0.010, *p* = 0.92. Percentage of correctness (correct/attempts) for nine boxes test Fe *n =* 97, MMN *n =* 87, *F* (*1,180*) = 1.830, *p* = 0.18. Aggregate score for the theory of mind (false belief task) Fe (*n =* 97), MMN (*n =* 87); no effect by group *F* (*1, 180*) = 0.771, *p* = 0.38, non-significant effect by gender *F* (*1, 180*) = 0.730, *p* = 0.39. Magnitude of Eta square (η^2^) Effect Size: 0.01 (small effect), 0.14 (Large effect). Effect Size: Cohen’s *d* ≤ 0.20 is a small effect.

#### 3.1.2. Nine Boxes

For the 9-boxes test, the 2 × 2 ANOVA revealed no main effect of group (*F(1, 180)* = 0.018, *p* = 0.89) or gender (*F*(*1, 180*) = 0.308, *p* = 0.58). There was also no significant group × gender interaction (*F(1, 180)* = 0.425, *p* = 0.52). The 2 × 2 ANOVA for percentage of correctness showed no group main effect (*F(1, 180)* = 1.830, *p* = 0.18), no main effect of gender (*F(1, 180)* = 0.893, *p* = 0.35), and no group × gender interaction (*F(1, 180)* = 0.437, *p* = 0.51).

#### 3.1.3. Theory of Mind (False Belief Task)

The 2 × 2 ANOVA conducted on data from the theory of mind test as an aggregate score yielded no main effects of group (*F(1, 180)* = 0.771, *p* = 0.38) or gender (*F(1, 180)* = 0.730, *p* = 0.39) or group ×gender interaction (*F(1, 180)* = 1.406, *p = 0.24*). Because the task seemed to be particularly hard for the children in the study, we examined the correlation between age and theory of mind performance.

Understanding of false belief seems to appear at 4.6 years old [64] and it is widely accepted that by 4 years of age most normally developing children have acquired an understanding of mind [66]. We found that there was a positive significant Pearson correlation between theory of mind and age in older children *r* (185) =0.18, *p* = 0.01, older children tended to perform better on the theory of mind test than children at a younger age within this Peruvian sample.

### 3.2. Social Emotional Functioning

The results from the ANOVAs for the BITSEA questionnaire are summarised in Table 3. For the BITSEA/P test, differences in problem scores between the Fe and the MMN were not significant (*F*(*1, 180*) = 1.570, *p* = 0.21). However, mothers with male children reported them to have had significantly more problematical behaviour that mothers whose children were girls, (*F*(*1, 180*) = 6.708, *p* = 0.01). There was no group × gender interaction effects for problematic behaviour as reported by the BITSEA (*F*(*1, 180*) = 1.582, *p* = 0.21). For the BITSEA/C test, ANOVA revealed no main effect of supplementation group (*F*(*1, 180*) = 0.067, *p* = 0.80) and no group × gender interaction (*F*(*1, 180*) = 0.041, *p* = 0.84). ANOVA did show that mothers with female children scored them higher on competence than mothers who had male children (*F*(*1, 180*) = 10.331, *p* < 0.01).

**Table 3:** Behaviour outcome: BITSEA test, problem and competence scores, by group and gender.

BITSEA	Fe (*n* = 97 )			MMN(*n* = 87)			*p* value	Eta squared η^2^	Cohen’s *d*	Effect size *r*
	$\bar{\text{x}}$ ± SD	Min.	Max.	$\bar{\text{x}}$ ± SD	Min.Max.					
**Problem**										
**All children**	**14.13 ± 5.99**	**3**	**33**	**15.17 ± 6.31**	**4**	**33**	**0.21**	**0.008**	**−0.26**	**−0.12**
Boys	15.70 ± 5.85 *	5	33	15.69 ± 6.78 *	4	33	0.01	0.035		
Girls	12.25 ± 5.65	3	29	14.50 ± 5.67	4	28				
**Competence**										
**All children**	**16.37 ± 3.22**	**7**	**22**	**16.45 ± 2.54**	**9**	**22**	**0.80**	**0.0004**	**−0.03**	**−0.014**
Boys	15.79 ± 2.99	7	21	15.82 ± 2.63	9	22		0.05		
Girls	17.07 ± 3.37 *	8	22	17.26 ± 2.19 *	13	22	<0.01			

Fe: boys (*n =* 53), girls (*n =* 44). MMN: boys (*n =* 49), girls (*n =* 38). * Significant differences by gender by ANOVA. Magnitude of Eta squared (η^2^) Effect Size: 0.01 (small effect), 0.06 (medium effect), 0.14 (Large effect). η_p_^2^ = partial eta squared. Problem score: *F* (*1, 180*) = 1.570, *p* = 0.21, η_p_^2^ = 0.009; η^2^ =0.008. There were significant differences by gender, where boys had higher problem scores than girls, *F* (*1, 180*) = 6.708, *p* = 0.01, η_p_^2^ = 0.036, η^2^ =0.035. Competence score: *F* (*1, 180*) = 0.067, *p* = 0.80, η_p_^2^ = 0.001, η^2^ =0.0004.There were significant differences by gender, such that the average competence in girls was significantly higher than boys, *F* (*1, 180*) = 10.331, *p* < 0.01, η_p_^2^ = 0.054, η^2^ =0.05.

On the BITSEA, mothers are also asked to consider whether their child behaviour represents a problem. For this question, ANOVA showed no main effects of supplementation group (*F*(*1, 180*) = 0.002, *p* = 0.96) main effects of gender (*F*(*1, 180*) = 4.861, *p* = 0.03), and no group × gender interaction (*F*(*1, 180*) = 0.018, *p* = 0.89). On the BITSEA, mothers were also asked to think about their child’s language development. ANOVA revealed no significant main effects of group (*F*(*1, 180*) = 1.468, *p* = 0.23) or a group × gender interaction (*F*(*1, 180*) = 0.088, *p* = 0.77). However, there were significant gender effect (*F*(*1, 180*) = 12.510, *p* < 0.01), with mothers reporting more concern about their child’s language development if their child was male than if their child was female.

#### WPPSI

The mean scores for each of the four subtests from the executive scale, and for each of the five subtests of the verbal scale are shown in Table 4. The sum of the scores for each scale was used to determine the Executive IQ and Verbal IQ, and from this Total IQ score. These are summarised in Table 5.

**Table 4:** WPPSI Executive and Verbal scores.

	Fe(*n* = 93)		MMN(*n* = 81)		*p* value	Maximum points (Number of questions)
	**$\bar{\text{x}}$ ± SD**		**$\bar{\text{x}}$ ± SD**			
	Boys (*n* = 52)	Girls (*n* = 41)	Boys (*n* = 44)	Girls (*n* = 37)		
**Executive subtests**						
Object assembly	8.10 ± 3.27	7.78 ± 3.30	8.09 ± 3.24	8.49 ± 2.99	0.47	32 (6)
Picture Completion	9.06 ± 2.82	8.90 ± 2.62	9.25 ± 2.54	9.14 ± 2.57	0.96	28 (28)
Block design	10.06± 2.49	9.88 ± 2.26	9.98 ± 2.67	10.54 ± 2.82	0.34	42 (14)
Animal Peg	9.17 ± 3.20	9.66 ± 3.34	9.41 ± 3.24	9.84 ± 3.43	0.96	70 (28 pegs)
**Verbal subtests**						
Comprehension	6.78 ± 1.90	6.80 ± 1.52	6.95 ± 1.68	6.75 ± 1.92	0.69	30 (15)
Vocabulary	5.88 ± 1.86	5.78 ± 1.59 **	5.68 ± 1.85	6.83 ± 2.05 **	0.03 **	47 (25)
Similarities	7.41 ± 2.69	7.72 ± 2.43	7.43 ± 2.50	7.86 ± 2.95	0.89	28 (12)
Arithmetic	8.57 ± 2.27	8.08 ± 2.60	7.75 ± 2.63	7.97 ± 2.70	0.36	23 (23)
Sentences	6.82 ± 2.67	7.95 ± 2.95 *	7.20 ± 2.48	7.83 ± 2.87 *	0.04 *	37 (12)

* Significant differences by gender by ANOVA. ** Significant differences by supplemented group by ANOVA.

For the executive IQ scores, a two-way ANOVA analysis revealed no main effects of group (*F*(*4, 167.0*) = 0.179, *p* = 0.95) or gender (*F*(*4, 167.0*) = 0.495, *p* = 0.74), and no group × gender effects (*F*(*4, 167.0*) = 0.465, *p* = 0.76). For the verbal IQ scores, ANOVA showed no main effects of group (*F*(*5, 163.0*) = 1.237, *p* = 0.29) and no group ×gender effects (*F*(*5, 163.0*) = 2.206, *p* = 0.06), but there was gender effect (*F*(*5, 163.0*) = 2.271, *p* = 0.05). There was significant group ×gender interaction for the “vocabulary” subtest that measured language development (*p* < 0.03). Girls in the MMN group had higher scores in the vocabulary test than girls in the Fe group. In addition, ANOVA showed that there was also a significant difference by gender for the verbal IQ subtest: “sentences” (*p* = 0.04), where girls had higher scores than boys. However, on application of the Bonferonni correction, neither of these differences remained statistically significant. All other verbal subtests scores showed no significant effect *p* > 0.05. For the total IQ scaled score the 2 × 2 ANOVA analysis yielded no main effect of group *(F*(*1, 170*) = 0.504, *p* = 0.48), no main effect of gender (*F*(*1, 170*) = 0.663, *p* = 0.42), and no group × gender interaction (*F*(*1, 170*) = 0.273, *p* = 0.60). There was a significant correlation between age and total WPPSI IQ scale, Pearson’s *r* (182) = 0.33, *p* < 0.0001.

**Table 5:** WPPSI Intelligent Quotient (IQ) by group.

	Fe (*n* = 93)	MMN (*n* = 81)	Mean difference	*p* value
	$\bar{\text{x}}$ ± SD	$\bar{\text{x}}$ ± SD		
**Executive IQ**				
**All children**	**93.95 ± 14.80**	**94.48 ± 16.75**	**0.53**	**0.87**
Boys	93.67 ± 14.65	95.48 ±13.78	1.81	
Girls	94.29 ±15.16	93.30 ± 19.86	0.99	
**Verbal IQ**				
**All children**	**83.31 ± 9.86**	**83.38 ± 11.09**	**0.07**	**0.92**
Boys	83.12 ± 10.30	82.23 ± 10.31	0.89	
Girls	83.56 ± 9.40	84.76 ± 11.94	1.2	
**Total IQ score**				
**All children**	**86.61 ± 12.23**	**87.90 ± 12.91**	**1.29**	**0.48**
Boys	86.37 ± 12.02	86.73 ± 12.15	0.36	
Girls	86.93 ± 12.64	89.30 ± 13.79	2.37	

## 4. Discussion

This study was designed to investigate whether multiple micronutrient supplementation had a long-term impact on cognitive and socio-emotional development in pre-school Peruvian children, in comparison to children who received iron supplement alone.

We found that there were no differences in scores on tests of inhibition, working memory and theory of mind between children aged 36–48 months who were given Fe or MMN supplements for 6 months during infancy. There were also no differences between the supplement groups in behavioural scores given by mothers. We did find gender differences across the two groups, in line with previous research [67], with boys scoring higher on problem behaviour and girls scoring higher on social competency.

Regarding other micronutrients necessary for brain development, there is iodine, but in our study iodine was not included in the supplement. In Peru, population iodine status is adequate and the control of iodine deficiency has been sustained for 30 years, since the implementation of the National Program for the Control of Endemic Goiter and Cretinism in 1985 [68].

With reference to zinc deficiency in Peru, the report by UNICEF and the International Zinc Association [69] states that although there are no specific statistics on zinc deficiency in Peru, the fact that zinc deficiency is linked to stunting and anaemia may suggest that zinc deficiency is high in children under 3 years old. About 41.6% of Peru’s population is at risk of inadequate zinc intake, and 18.3% of children under 5 years old are stunted [69]. In the capital Lima, the percentage of anemic children is 37.7% in children aged from 6–36 months old [70].

Regarding the impact of MMN on biomarkers of status in the original cohort study, after 6 months of supplementation, there was a positive effect in reducing anaemia in both groups and there was an improvement of zinc status in the multiple micronutrients group [71].

To date, there have been few studies that examine the impact of multiple micronutrient supplements on long term cognitive development in children. In terms of single micronutrient supplement studies, Taneja, *et al.* [72] reported that zinc supplementation in young children aged 12–18 months did not affect the mental or psychomotor development index scores. The data from our study concur with this, since the MMN supplement contained zinc, and thus the iron alone supplement considered the “control” for the sake of this comparison. In other studies of micronutrient supplementation Murray-Kolb, *et al.* [73] found no long-term developmental benefits of iron plus folic acid, zinc alone, or iron plus folic acid supplementation given to children from Nepal from 12 to 35 months of age at supplementation. It is noteworthy that these children also received micronutrient supplementation in utero, through their mothers who were supplemented daily from early pregnancy through 3 months postpartum [74]. These Nepali children were then tested on intellectual, executive, and motor function at age 7–9 years of age. In contrast, the review by Black [4] indicated that children who were zinc supplemented demonstrated higher neuropsychological performance than the control. One of the studies reviewed was the study by Penland, where 372 children aged 6–9 years old were given either 20 mg of Zn, 20 mg of Zn with additional micronutrients (including iodine, selenium, copper, manganese, fluoride and folate) [75] or micronutrients without zinc, 6 days per week for 10 weeks. This study found that children who were given Zn or Zn with micronutrients performed better in visual memory (delayed design matching), tracking the cursor on the screen, continuous vigilance test, and perception (design matching task), than children who were given micronutrients alone [76]. Similarly the study by Sandstead also indicated that neuropsychological performance (tapping, circular tracking, and continuous vigilance tests) was enhanced when 740 children aged 6–9 years old were supplemented with zinc and micronutrients 6 days per week for 10 weeks, compared to zinc alone or micronutrients alone [77].

Both the Penland [76] and Sandstead [77] studies tested children after supplementation for 10 weeks and found positive improvement in neuropsychological performance, which does not concur with our study, possibly because the children were older (aged 6-7 years) compared with ours (aged 36–48 months old). Whereas the study by Murray-Kolb was a cohort follow-up study of children who received supplementation in utero and also when aged 12–35 months old, they were then tested aged 7–9 years old and found no significant improvement in cognitive function. Our study is similar to Murray-Kolb study [73] that when assessed pre-school children in the long term, found no positive effect of cognitive function.

The one significant difference between the iron and the MMN group was found on only one verbal IQ subtest, the vocabulary subtest, where girls in the MMN group had higher vocabulary scores than girls in the iron group. However, the significance did not hold when the Bonferroni correction was applied. Differences by gender were found in our study where girls scored higher than boys in the sentences subtest verbal IQ but this differences were not significant. Previous researchers have reported differences by gender such as the study by Palejwala [78] which indicated that girls aged 2 to 7 years old showed higher general intelligence, girls aged 4 to 7 years old performed better in processing speed, boys aged 4–7 performed better in visual processing but this was not noticed in younger boys aged 2 to 3 years old. The study by Quereshi in 72 children aged between 5–6 years, showed that boys had significantly (*p* < 0.05) higher mean verbal IQs than girls [79]. Liu [80] also found gender differences in WPPSI test scores among Chinese children aged 5–6 years old, where boys obtained higher IQ than girls. It may be that because children in our study were under 4 years old, girls appeared to have performed better in the WPPSI IQ verbal subtest sentences. Carlson [81], found a correlation between age and total IQ and this finding was similar to our study.

It may be important to consider the timing and length of supplementation, since brain growth during infancy and early childhood may have a greater potential for impact through diet than brain growth during foetal life [82] and zinc is critically important for cognitive development during early childhood [83]. A recent review and meta-analysis showed that supplementation during pregnancy had little impact on indices of cognitive development [84], whereas supplementation given to the children did have a positive impact on two domains namely executive and motor function. Most studies have focused on the impact of nutrition on cognition in children under 2 years old, but spurts of brain development continue during childhood (7–9 years old) and through adolescence [85]. Therefore, the timing of nutritional influences on the brain is important but remains complex [86], for instance the brain is particularly sensitive to nutritional deficiencies between 24–42 weeks of gestation where neurological processes occur such as synapse formation and myelination [87]. Furthermore, the first two years of life is the period of most rapid brain growth, at 2 years the brain reaches 80% of its adult weight [88], and at 5 years, the brain size is about 90% of its adult size [89]. What remains widely accepted is that intervention during the first 1000 days of life is crucial to break the cycle of malnutrition [90].

A systematic review on prenatal micronutrient supplementation on children’s mental development found that interventions were inconsistent and inconclusive, but there was some evidence to support a positive effect of n-3 fatty acids or multi-micronutrients on mental development [91]. In addition, MMN supplementation given to pregnant women improved their children’s mental development at 1 year old [92] and children’s motor and cognitive abilities up to 3.5 years old when MMN supplements were given to undernourished or anaemic mothers [93].

The main strength of this study was that the psychological tests provided robustness of aspects of cognitive function and the assessment of diverse outcomes from different perspectives, encompassing a wide range of test processes. The cut-off points developed for BITSEA for children slightly older than in previous studies would likely be of use elsewhere.

A limitation of the study was that since the original study was not designed to assess cognitive function, the micronutrient supplement composition was not specifically selected for this health outcome. Other micronutrients are important but not all were included in the multiple micronutrient supplementation.

Another limitation of the study was that although the children were randomly selected from the original study cohort, the parents were given the option of participating or not which may have introduced some selection bias. We also need to note, that some tests had not been used on Peruvian children before, and the appropriateness of the cross-cultural aspects of psychological assessment [94] have not being examined in the current study. The theory of mind task appeared to be particularly difficult, although we did find that older children in the sample were better at this task than the younger children, which fits with previous research showing that theory of mind, as defined by false belief, is often not intact at 3 years and children tend to fail this task [66], but is at 4 years, where children appear to have a better understanding of false belief [64].

## 5 Conclusions

Our study found that there were no long-term impacts of MMN supplementation on cognitive outcomes of executive function, intelligence, and behaviour when compared to iron supplementation alone. Expected differences between boys and girls on cognitive and behavioural competence scales were found. Further research is necessary to investigate the effect of MMN supplementation on cognitive function and the optimal combination of supplementation, particularly for the most deprived populations at high risk of micronutrient deficiencies. It is also important to study the timing and length of nutrition supplementation, in order to identify the optimal period for intervention for maximal benefit on the cognitive development of a child, either pre-or post-nataly.

## Abbreviations

BITSEA: Brief Infant Toddler Social Emotional Assessment
BITSEA/P: BITSEA Problem total score
BITSEA/C: BITSEA Competence total score
Fe: Iron supplemented group
IQ: Intelligence Quotient
IIN: Instituto de Investigación Nutricional
MMN: Multiple micronutrient (including zinc) supplemented group
RCT: Randomized control trial
RDA: Recommended Daily Allowance
SD: Standard Deviation
UCLAN: University of Central Lancashire
UNICEF: United Nations Children’s Fund
WPPSI: Wechsler Preschool and Primary Scale of Intelligence
WPPSI -R: Wechsler Preschool and Primary Scale of Intelligence Revised
